# 4258 Black women’s narratives: A mixed-methods exploration of microaggressions and mental health

**DOI:** 10.1017/cts.2020.261

**Published:** 2020-07-29

**Authors:** Alexandria Colburn, Ed de St. Aubin

**Affiliations:** 1 Marquette University

## Abstract

OBJECTIVES/GOALS: This social justice-oriented, multi-method study aims to gain an understanding of the unique sources of stress and resilience impacting Black women in Milwaukee. As clinical researchers, it is imperative that we understand the mechanisms underlying the relationship between marginalized identities and substantial health disparities. METHODS/STUDY POPULATION: Participants were Black women, diverse in age, income, and sexual orientation to emphasize an intersectional approach (current N = 87 of 160). Our interdisciplinary team collected two interrelated data types: narrative and survey. Participants completed a 1.5-hour life story interview in which they were asked to share stories from their lives, their backgrounds, plans for the future, forces that shaped their stories, and how their identities have influenced their experiences. Interviews were done one-on-one and conducted by Black women interviewers. They were also asked to complete an online survey protocol including measures of stress, trauma, microaggressions, coping, and well-being. Transcribed life story interviews will be coded utilizing grounded theory, an intensive qualitative analysis method. RESULTS/ANTICIPATED RESULTS: The presentation will focus on the unique methodological approach, which emphasizes community empowerment through collaboration and cultural competency. Data collection is still in progress, but initial relationships between key variables will be discussed. It is anticipated that greater frequency and appraisal of microaggressions will significantly predict higher reported stress, anxiety, and depression. Within-group diversity will be examined as well. Relevant themes emerging from grounded theory will also be presented. Results will directly inform community outreach aimed at improving the lives of Black women. DISCUSSION/SIGNIFICANCE OF IMPACT: This study sheds light on unique, identity-related stressors believed to contribute to mental and physical health disparities. It also builds on current theories, filling voids in the research literature by taking a psychosocial perspective, emphasizing the voices of participants, and channeling the findings to specific programs in the community.

